# Extended Hellwig’s Method Utilizing Entropy-Based Weights and Mahalanobis Distance: Applications in Evaluating Sustainable Development in the Education Area

**DOI:** 10.3390/e26030197

**Published:** 2024-02-25

**Authors:** Ewa Roszkowska, Marzena Filipowicz-Chomko, Anna Łyczkowska-Hanćkowiak, Elżbieta Majewska

**Affiliations:** 1 Faculty of Computer Science, Bialystok University of Technology, Wiejska 45A, 15-351 Bialystok, Poland; e.roszkowska@pb.edu.pl; 2 Institute of Economics and Finance, WSB Merito University in Poznań, Ul. Powstańców Wielkopolskich 5, 61-895 Poznań, Poland; anna.lyczkowska-hanckowiak@poznan.merito.pl; 3 Faculty of Economics and Finance, University of Bialystok, Warszawska 63, 15-062 Bialystok, Poland; e.majewska@uwb.edu.pl

**Keywords:** multi-criteria decision making, Hellwig’s method, entropy-based weights, Mahalanobis distance, Euclidean distance, sustainable development, education

## Abstract

One of the crucial steps in the multi-criteria decision analysis involves establishing the importance of criteria and determining the relationship between them. This paper proposes an extended Hellwig’s method (H_EM) that utilizes entropy-based weights and Mahalanobis distance to address this issue. By incorporating the concept of entropy, weights are determined based on their information content represented by the matrix data. The Mahalanobis distance is employed to address interdependencies among criteria, contributing to the improved performance of the proposed framework. To illustrate the relevance and effectiveness of the extended H_EM method, this study utilizes it to assess the progress toward achieving Sustainable Development Goal 4 of the 2030 Agenda within the European Union countries for education in the year 2021. Performance comparison is conducted between results obtained by the extended Hellwig’s method and its other variants. The results reveal a significant impact on the ranking of the EU countries in the education area, depending on the choice of distance measure (Euclidean or Mahalanobis) and the system of weights (equal or entropy-based). Overall, this study highlights the potential of the proposed method in addressing complex decision-making scenarios with interdependent criteria.

## 1. Introduction

Multi-criteria decision-making (MCDM) is an important field in operations research and decision analysis, providing structured approaches to handle complex decision problems involving multiple, often conflicting, criteria [1,2,3]. The motivation for this study is related to the assessment of criteria weights and whether they are independent or dependent on one another [4,5,6,7,8] within the multi-criteria procedure. This issue plays a critical role in shaping the decision process and the outcome [8,9,10]. Understanding and appropriately modeling the importance of criteria and the relationships between them are key elements for making effective and informed decisions in a wide range of applications [9,11,12]. 

Considering the motivation, the objectives of this study are twofold. Firstly, we propose an extension of the Taxonomic Measure of Development established by Hellwig [13]. Hellwig’s method is recognized as particularly useful within the field of economic research [14,15,16]. It relies on the concept of distance to the ideal (pattern of development). In the original approach, equal weights are assumed and distances are calculated using Euclidean distance, assuming implicitly that the considered criteria are independent. However, in real-world situations, the assumption of criteria independence is rarely met.

In this extension, we utilize entropy-based weights and Mahalanobis distance. Incorporating the concept of entropy allows for the effective assignment of weights to criteria based on their information content, thereby reducing the subjectivity in weight assignment. Higher entropy values imply greater uncertainty, leading to a higher weight, as the criterion carries more decision-relevant information. Conversely, lower entropy indicates a more predictable criterion, resulting in a lower weight. Therefore, entropy provides an objective method for determining criterion weights. By addressing uncertainty through entropy, they enable more robust decision-making, particularly in situations with incomplete or ambiguous information. Incorporating Mahalanobis distance allows for taking into account the interdependencies among criteria. This modification aims to enhance the accuracy and robustness of multi-criteria decision-making (MCDM) processes, especially in situations where criteria are not independent.

The second objective of this paper is to examine the relevance and effectiveness of the proposed extended Hellwig’s method (H_EM) in practice. The H_EM method has been applied to assess the achievement of Sustainable Development Goal 4 (SDG4) of the 2030 Agenda by the European Union countries in the field of education in 2021. In this study, we utilized data provided by Eurostat for the year 2021 concerning the Sustainable Development indicators related to education (SDG 4) for EU member states. The SDG 4 set consisting of five indicators comprises the main aspects designed to track progress across various educational levels and domains.

Furthermore, a performance comparison was conducted between the extended Hellwig’s method and other versions of Hellwig’s approaches. The findings suggest that the correlation between the criteria, specifically the choice of distance measure (Euclidean or Mahalanobis) and the system of weights (equal or entropy-based), significantly influences the ranking of the EU countries in education while using different versions of Hellwig’s method.

This paper follows the structure outlined below: Section 2 introduces the classical Hellwig’s method and its extension through a new distance measure and entropy-based weights. In Section 3, the goals in the education area, the empirical data used, and the results obtained are presented. Section 4 offers a comparative analysis of the research findings, particularly the rank-ordering of the EU countries achieved through the proposed techniques, in contrast to the results obtained by the other variants of Hellwig’s method. This paper is rounded off with a summary in the concluding remarks. 

## 2. The Classical Hellwig’s Method and Its Extensions

Hellwig’s multi-criteria method, also known as the Taxonomic Measure of Development, is a ranking technique used in multi-criteria analysis [13]. This method was developed by Zdzisław Hellwig, a Polish economist and mathematician, in the year 1968 for evaluating and comparing countries with respect to the level of development and the resources and structure of qualified personnel. This method is based on the measurement of the Euclidean distance of each object from the reference object, the so-called ideal or development pattern. 

The classical Hellwig’s method, as described in [13], has undergone various modifications to deal with real data [17], as well as extensions to fuzzy sets [18], intuitionistic fuzzy sets [19,20,21,22], and interval-valued fuzzy sets [23]. The classical Hellwig’s method and its modifications or extensions have found applications in the analysis of complex socio-economic phenomena across a wide range of domains, including but not limited to the circular economy [15], quality of human capital in the EU countries [14], socio-economic region development [18], sustainable development [24], quality of life [20,21], and evaluation negotiation offers [22,25].

It is worth noticing that Hellwig’s technique is close to the TOPSIS (Technique for Ordering Preferences by Similarity to Ideal Solution) procedure, which is often applied in the MCDM area [26]. Hellwig’s measure of economic development has been proposed for linear ranking in the field of economics for analyses of complex socio-economic phenomena, while the TOPSIS method has been proposed for linear ranking in the field of decision theory (MCDM). Both methods are frequently used for empirical research to establish the order of objects described by a set of variables. The TOPSIS procedure uses the concept of distances to the ideal and ant-ideal, while Hellwig’s method uses only the concept of distances to the ideal (pattern development).

### 2.1. Classical Hellwig’s Method

The original Hellwig’s method [13] orders the alternatives according to the distances from the ideal one. In multi-criteria methods, alternatives are the various options or choices under evaluation, and criteria are the specific factors or attributes used to assess these alternatives. The selection of alternatives and criteria depends on the context of the decision or problem. In situation analyses of social phenomena, the alternatives are objects, for e.g., countries, cities, and regions, and criteria are individual indicators, for e.g., sustainable development indicators from Eurostat and indicators from public statistics characterizing the phenomenon. First, we have to collect and organize the data, which may include information about different alternatives and their performance across multiple criteria.

Suppose that we have m alternatives $A_{1},A_{2},\ldots ,A_{m}$ and n decision criteria $C_{1},C_{2},\ldots ,C_{n},$ where $x_{ij}$ denotes the criteria value of $A_{i}$ on $C_{j}\;$(i=1,2,…,m; j=1,2,…,n). 

The classical Hellwig’s method [13] with the additional step concerning determination weights can be summarized as follows:

Step 1. Determining the decision matrix

(1) $$D=\left[x_{ij}\right],$$

where $x_{ij}$ is the value of the j-th criterion for i-th alternative i=1,…,m, j=1,…,n.

Step 2. Defining the vector of weights

(2) $$W=\left[w_{1},\ldots ,w_{n}\right],$$

where $w_{j}>0\;(j=1,\ldots ,n)$ is the weight of the criterion $C_{j}$ and $\sum_{j=1}^{n}w_{j}=1$.

Step 3. Building the ideal (pattern of development)

(3) $$I=\left[x_{1}^{+},\ldots ,x_{n}^{+}\right],$$

where

(4) $$x_{j}^{+}=\left\{\begin{array}{l} \underset{i}{\max}x_{ij}\;for\;benefit\;criterion\\ \;\underset{i}{\min}x_{ij}\;for\;cost\;criterion. \end{array}\right.$$

Step 4. Building the normalized matrix

(5) $$\bar{D}=\left[{\bar{x}}_{ij}\right],$$

where

(6) $${\bar{x}}_{ij}=\frac{x_{ij}-\bar{x_{j}}}{S_{j}},$$

and $\bar{x_{j}}=\frac{1}{m}\sum_{i=1}^{m}x_{ij}$, $S_{j}=\sqrt{\frac{1}{m}\sum_{i=1}^{m}{\left(x_{ij}-\bar{x_{j}}\right)}^{2}}$.

We standardize the data by subtracting the mean and dividing it by the standard deviation for each criterion. This step is crucial to ensure that all criteria are on the same scale.

Step 5. Building the weighted normalized matrix

(7) $$\tilde{D}=\left[{\tilde{x}}_{ij}\right],$$

where

(8) $${\tilde{x}}_{ij}=w_{j}{\bar{x}}_{ij}.$$

Step 6. Calculating the distances ${(d}_{i0})$ of i-th alternative $A_{i}$ from the ideal I by using classical Euclidean distance measure

(9) $$d_{i0}(A_{i},I)=E({\tilde{A}}_{i},\tilde{I})=\sqrt{\sum_{j=1}^{n}{\left({\tilde{x}}_{ij}-{\tilde{x}}_{j}^{+}\right)}^{2}}$$

Step 7. Calculating Hellwig’s measure $H_{i}$ for the i-th alternative as follows

(10) $$H_{i}=1-\frac{d_{i0}}{d_{0}},$$

where $d_{0}=\bar{d}+2S,$ for $\bar{d}=\frac{1}{m}\sum_{i=1}^{m}d_{i0}$, $S=\sqrt{\frac{1}{m}\sum_{i=1}^{m}{{(d}_{i0}-\bar{d})}^{2}}.$

Step 8. Ranking of alternatives according to descending $H_{i}$.

$H_{i}$, as determined following the described procedure, is a normalized measure typically ranging from zero to one. A greater value of the synthetic measure corresponds to a higher ranking position for the respective alternative.

### 2.2. Entropy-Based Weights Method

The entropy-based weights method is a technique commonly employed in MCDM to determine the relative importance of criteria [26,27,28,29,30,31]. It is founded on the principle of information entropy, which measures the uncertainty associated with a set of data [32]. Entropy-based weighting has gained significant attention in recent years due to its ability to offer an objective way to calculate weights. The calculation is based on the data available in the matrix, which reduce the influence of subjective judgments [33,34,35,36]. This is particularly valuable in decision-making processes where transparency and fairness are essential challenges related to subjective weighting. 

Let $D=\left[x_{ij}\right]$ be the decision matrix, where $x_{ij}$ is the value of the j-th criterion for i-th alternative i=1,2,…,m, j=1,2,…,n. The weight of j-th criterion can be calculated as follows [26]:

(11) $$w_{j}=\frac{1-E_{j}}{\sum_{j=1}^{n}\left(1-E_{j}\right)}=\frac{1-E_{j}}{n-\sum_{j=1}^{n}E_{j}},\;j=1,2,\ldots ,n,$$

where $E_{j}$ is an extended and normalized entropy defined as follows:

(12) $$E_{j}=-\frac{1}{lnm}\sum_{i=1}^{m}\frac{x_{ij}}{\sum_{i}^{m}x_{ij}}ln\frac{x_{ij}}{\sum_{i}^{m}x_{ij}},\;j=1,2,\ldots ,n.$$

It is easy to check that ${0\leq w}_{j}\leq 1\;(j=1,\ldots ,n)$ and $\sum_{j=1}^{n}w_{j}=1$ according to the properties of entropy.

It is also worth noting that in some studies decision matrix D is normalized before applying the Formula (12). Chen [27] has investigated the impact of normalization on the entropy-based TOPSIS method. The entropy-based weights method relies on the principle that criteria with higher entropy (greater variability) are considered more important. Conversely, criteria with lower entropy are perceived as less influential in the decision-making process. The entropy-based weights method is particularly useful in cases where there is limited a priori knowledge about the criteria’s relative importance, making it a valuable tool for unbiased decision support. Numerous studies have explored and applied this method in various fields, such as management [37], finance [38], environmental quality [39], sustainable energy [35,40], water resources management [9], location selection [41], urban air quality [42], and tourism [40,43].

### 2.3. Mahalanobis Distance in Decision Making

The Mahalanobis distance, introduced by Mahalanobis in 1936 [44], is a statistical measure of distance that is particularly useful in classification, clustering, and multi-criteria decision-making. The Mahalanobis distance measures the distance between two points in a multi-dimensional space while accounting for the covariance between the criteria. The covariance matrix represents the relationships and dependencies between the criteria taking into account the correlation between the criteria measuring the distance between two points. When the covariance matrix is equal to the identity matrix, the Mahalanobis distance simplifies to the Euclidean distance. 

The weighted Mahalanobis distance between points $x=(x_{1},\;x_{2},\ldots ,x_{n})$, *y*$=(y_{1},\;y_{2},\ldots ,y_{n})$ is computed using the following equation [35,45]:

(13) $$M(x,y)=\sqrt{(x-y)W\;C^{-1}{W^{T}(x-y)}^{T}},$$

where C is the variance–covariance matrix of data matrix D with m objects in rows by n columns, and $W=diag(\sqrt{w_{1}},\ldots ,\sqrt{w_{n}})$ is the diagonal matrix, where $w_{1},\;w_{2},\ldots ,w_{n}$ are the weights assigned to the criteria. 

For more detailed computation information and a comparative analysis of Euclidean distance and Mahalanobis, see [46]. 

The Mahalanobis distance can be used in various MCDM methods, such as TOPSIS [10,35,45,47,48,49], TODIM (an acronym in Portuguese for Interactive and Multi-criteria Decision Making) [50], or other decision-making problems [51,52,53,54]. It helps DMs identify the most suitable alternative according to their preferences and objectives while considering the multi-dimensional nature of the data and the interplay between criteria. 

### 2.4. Normalization Formulas

The normalization process represents a crucial stage in the majority of MCDM methods. It serves to convert input data, which may be expressed in varying units, into numerical and comparable values. The literature [1,26,55] has introduced numerous normalization techniques that can be employed within MCDM approaches for the evaluation and ranking of alternatives. Jahan and Edwards [55] defined thirty-one normalization techniques, scrutinized their limitations, and suggested enhancements for their application in the decision-making process of engineering design. These normalization techniques are mainly categorized into three classes: vectors, linear transformations, and non-linear transformations. Later, a comparative analysis of six well-established normalization techniques in the context of MCDM problems was conducted.

The impact of different normalization procedures on the ranking of alternatives obtained by MCDM methods has been studied by many authors [56,57,58,59,60,61,62,63]. Chakraborty and Yeh [57] compared the same normalization procedures within the TOPSIS method and found that vector normalization consistently yielded the most reliable results across different problem sizes, while linear max-min normalization was the least consistent. Vafaei et al. [62] evaluated six normalization techniques in the context of the TOPSIS method. Using Pearson and Spearman correlation coefficients, they concluded that the most suitable normalization technique for the TOPSIS method is vector normalization. Hellwig’s method belongs to the same group of multi-criteria methods based on an aggregation formula as TOPSIS. Therefore, we will apply a vector normalization formula in constructing our synthetic measure. 

For the decision matrix $D=\left[x_{ij}\right],$ where $x_{ij}$ is the value of the j-th critierion for i-th alternative i=1,2,…,m, j=1,2,…,n, the normalized value ${\bar{x}}_{ij}$ is obtained by divided performance rating $x_{ij}$ by its norm, shown as follows:

(14) $${\bar{x}}_{ij}=\left\{\begin{array}{c} \frac{x_{ij}}{\sqrt{\sum_{i=1}^{m}{\left(x_{ij}\right)}^{2}}}\;for\;benefit\;criterion,\\ \frac{1/x_{ij}}{\sqrt{\sum_{i=1}^{m}{\left({1/x}_{ij}\right)}^{2}}}\;for\;cost\;criterion, \end{array}\right.$$

where $x_{ij}$ is the value of the j-th criterion for i-th alternative i=1,2,…,m, j=1,2,…, n.

### 2.5. Extended Hellwig’s Method Utilizing Entropy-Based Weights and Mahalanobis Distance

The extended Hellwig’s method utilizing entropy-based weights and Mahalanobis distance can be summarized as follows. Modifications have been implemented in Step 2, Step 4, and Step 6 of the classical Hellwig’s procedure (see Section 2.1).

Step 1. Determining the decision matrix $D=\left[x_{ij}\right],$ where $x_{ij}$ is the value of the j-th criterion for i-th alternative (i=1,2,…,m, j=1,2,…,n).
Step 2. Determining the weight vector $W=\left[w_{1},\ldots ,w_{n}\right]$ using Equations (11) and (12).
Step 3. Building the ideal $I=\left[x_{1}^{+},\ldots ,x_{n}^{+}\right]$ using Equation (4).
Step 4. Building the normalized matrix $\bar{D}=\left[{\bar{x}}_{ij}\right]\;(i=1,2,\ldots ,m$, j=1,2,…,n) using Equation (14).
Step 5. Calculating the distances ${(d}_{i0})$ of i-th alternative $A_{i}$ from the ideal I by using the Mahalanobis distance measure (Formula (13)) as follows:
  (15) $$d_{i0}(A_{i},I)=M({\bar{A}}_{i},\bar{I})=\sqrt{({\bar{A}}_{i}-\bar{I}){WC}^{-1}{W^{T}({\bar{A}}_{i}-\bar{I})}^{T}},$$
  where *C* is the variance–covariance matrix of the data matrix $\bar{D},\;W=diag(\sqrt{w_{1}},\ldots ,\sqrt{w_{n}})$ is the diagonal matrix, where $w_{1},\;w_{2},\ldots ,w_{n}$ are the weights assigned to the criteria.

More precisely, we adopt the Mahalanobis distance formula between the alternative and ideal used for the TOPSIS method studied in the paper [45].

Step 6. Calculating the extended Hellwig’s measure ${H\_EM}_{i}$ for the i-th alternative using Formula (10).
Step 7. Ranking of objects according to descending ${H\_EM}_{i}$.

Let us observe that the extended Hellwig’s method replaces equal weights with an entropy-based system and incorporates the Mahalanobis distance measure instead of Euclidean distance to deal with dependencies between criteria.

## 3. An Empirical Case Study: Evaluating Sustainable Development in the Education Area with the Extended Hellwig’s Procedure 

The SDGs are a set of 17 global goals adopted by all United Nations Member States in 2015 as part of the 2030 Agenda for Sustainable Development [64]. These goals represent a critical aspect of the broader sustainable development agenda and are designed to address a wide range of global challenges and promote a more sustainable, equitable, and prosperous world by the year 2030 [64]. To assess and measure progress towards the realization of SDGs, multi-criteria methods offer a robust framework that considers diverse dimensions [65,66,67,68]. Those methods can be used for international comparisons of a country taking into account various indicators and assessment criteria and for monitoring progress in achieving SDGs in a specific country. They can be applied regularly to track changes in the realization of SDGs in different areas. The results of multi-criteria analyses can be also utilized to formulate and adjust a country’s policy to improve the progress and the attainment of SDGs.

### 3.1. Problem Description

Among the seventeen SDGs, SDG 4 is a significant agenda item, focusing on matters related to education [17,69]; SDG 4 is often referred to as “Quality Education”. Its full title is as follows:

Goal 4: *Ensure Inclusive and Equitable Quality Education and Promote Lifelong Learning Opportunities for All*.

SDG 4 recognizes the transformative power of education in achieving sustainable development [70,71]. Education is not only a fundamental human right but also a key driver in reducing poverty, improving health, fostering economic growth, and promoting peace and social cohesion. Efforts to achieve this goal involve not only increasing access to education but also improving its quality, relevance, and inclusivity. Governments, organizations, and communities worldwide are working towards realizing the vision of SDG 4 to ensure quality education and lifelong learning opportunities for all.

Two research questions can be asked in this context:

1. How do the different systems of weights (equal vs. entropy-based) affect the ranking of the EU countries obtained by Hellwig’s method?
2. How do different distance measures (Euclidean vs. Mahalanobis) affect the ranking of the EU countries obtained by Hellwig’s method?

### 3.2. The Source of Data 

In this study, we utilized data provided by Eurostat for the year 2021 concerning the Sustainable Development indicators related to education (SDG 4) for the EU member states. Table 1 presents five indicators that track progress towards SDG 4 in the EU countries in 2021. The SDG 4 indicator set comprises the main aspects designed to track progress across various educational levels and domains. For detailed definitions of each indicator, refer to [Eurostat SDG] [72]. 

**Figure 1:**
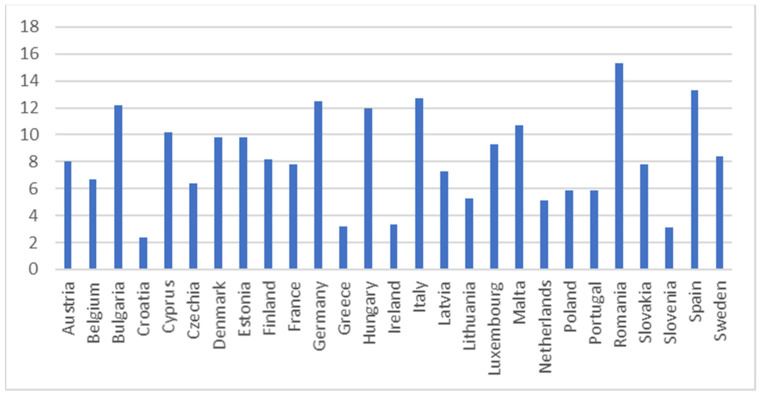
Early leavers from education and training (%). Source: Eurostat [sdg_04_10a].

**Figure 2:**
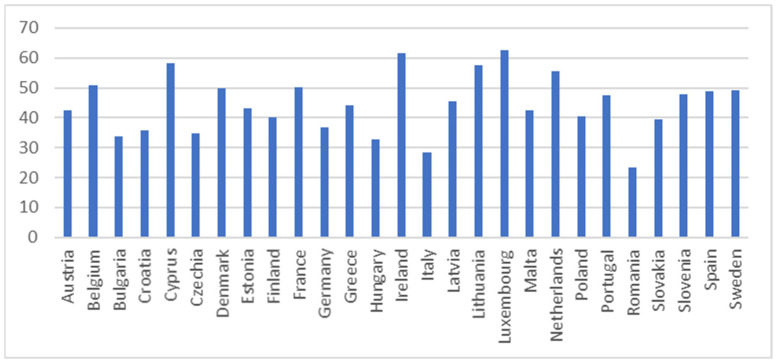
Tertiary educational attainment (%). Source: Eurostat [sdg_04_20].

**Figure 3:**
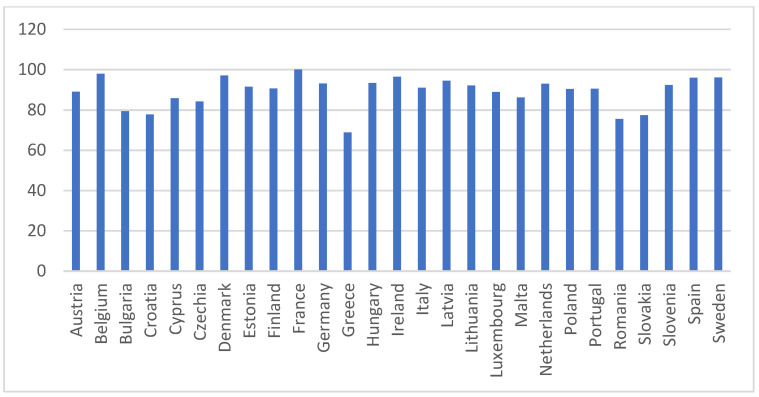
Participation in early childhood education (%). Source: Eurostat [sdg_04_31].

**Figure 4:**
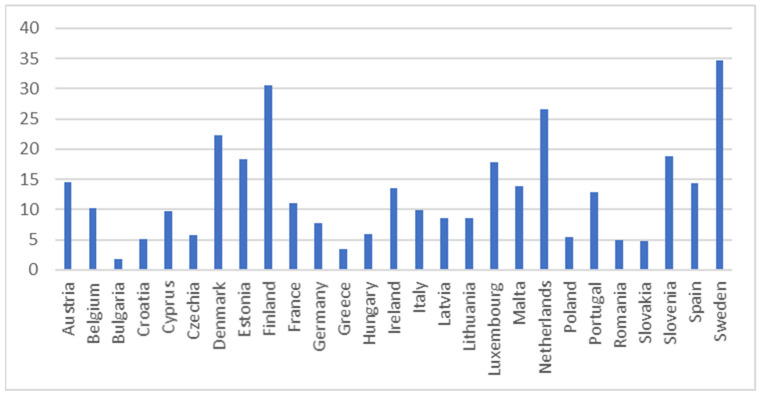
Adult participation in learning in the past four weeks indicator (%). Source: Eurostat [sdg_04_60].

**Figure 5:**
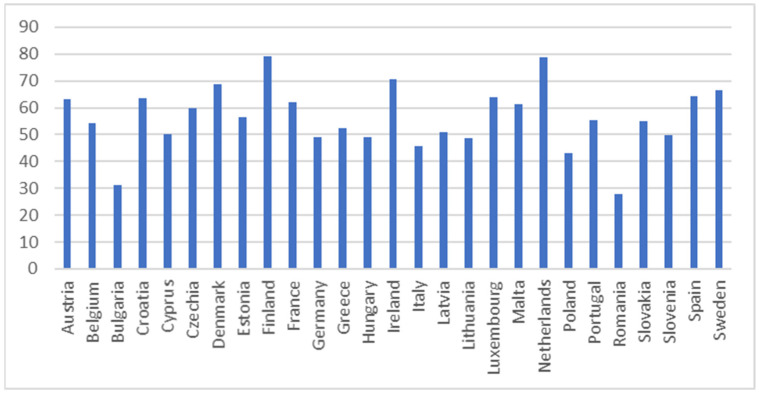
Share of individuals having at least basic digital skills indicator. Source: Eurostat [sdg_04_70].

The descriptive statistics for SDG 4 indicators in 2021 are presented in Table 2 along with related Figure 1, Figure 2, Figure 3, Figure 4 and Figure 5. 

The early leavers from education and training indicator assess the proportion of individuals aged 18 to 24 with a maximum of lower secondary education who did not participate in any education or training during the four weeks preceding the survey [72]. Figure 1 displays early leavers from education and training rates in the EU countries for 2021. This indicator showed a high variation (40.84%) compared to other indicators. The countries with the most concerning situation were Romania, Spain, and Italy. In contrast, the countries with the best results were Croatia, Slovenia, and Greece.

The tertiary educational attainment indicator assesses the percentage of individuals aged 25–34 who have attained a successful completion of tertiary studies, such as university or higher technical institutions [70]. According to Figure 2, the best tertiary educational situation was observed in Luxembourg, Ireland, and Cyprus, while the worst was in Romania, Italy, and Hungary. 

The participation in early childhood education indicator evaluates the percentage of children between three years old and the starting age of compulsory primary education who have engaged in early childhood education and care (ECEC), classified as ISCED level 0 according to the International Standard Classification for Education (ISCED 2011) [70]. Figure 3 presents the participation in early childhood education indicator in the EU countries for 2021. This indicator showed the least variation (8.40%) compared to the other indicators. The situation was the best in France, Belgium, and Denmark, but the worst in Greece, Romania, and Slovakia.

The adult participation in learning in the past four weeks indicator assesses the percentage of individuals aged 25 to 64 who reported undergoing formal or non-formal education and training in the four weeks preceding the survey (numerator). The denominator comprises the total population within the same age group, excluding those who did not respond to the question regarding participation in education and training. Adult learning encompasses both general and vocational formal and non-formal learning activities, typically occurring after the completion of initial education [70]. Figure 4 exhibits adult participation in learning in the past four weeks in the EU countries for 2021. This indicator depicts the greatest variation (64.73%) compared to the other indicators. The greatest participation of adults in learning were observed in Sweden, Finland, and the Netherlands. However, the lowest participation of adults in learning were noticed in Bulgaria, Greece, and Slovakia. 

The share of individuals having at least basic digital skills indicator measures the percentage of individuals aged 16 to 74 possessing a minimum proficiency in digital skills. This indicator is derived from specific activities individuals engage in on the internet, encompassing information and data literacy, communication and collaboration, digital content creation, safety, and problem-solving [70]. Figure 5 displays the share of individuals having at least a basic digital skill indicator in the EU countries for 2021. According to Figure 5, the highest percentages of individuals having at least basic digital skills were observed in Finland, the Netherlands, and Ireland, while the lowest were in Romania, Bulgaria, and Poland.

Monitoring SDG 4 within the European Union context emphasizes basic, tertiary, and lifelong education. The complete set of SDG 4 indicators provides insights into overall educational achievements and their influence on the job market. These allow us to examine various educational stages from early childhood education, encompass the development of fundamental skills (literacy, numeracy, and science proficiency), and culminate in tertiary education and continued adult learning participation.

### 3.3. Results 

This study evaluates the realization of SGD 4 for the EU countries in 2021 using the H_EM method. To assess the efficacy of the newly introduced H_EM method, this study initially employs the Pearson coefficient to investigate the correlation among the criteria. The results of this analysis are presented in Table 3, indicating a strong positive correlation between criteria $C_{4}$ and $C_{5}$ (0.706) and a moderate correlation between $C_{2}$ and $C_{5}$ (0.520) and between $C_{3}$ and $C_{4}$ (0.506). These findings justify the use of the H_EM method. It is also noteworthy that the negative Pearson coefficient between $C_{1}$ and criteria $C_{2}$, $C_{4}$, and $C_{5}$ further confirms the negative impact of criterion $C_{1}$ on education. However, the Pearson coefficient between $C_{1}$ and $C_{3}$ is positive, but very small (0.037) and not statistically significant. 

For comparative analysis, we employed three variants of the Hellwig’s method: (1) with equal weights and Euclidean distance (H_E), (2) with entropy-based weights and Euclidean distance (H_EE), and (3) with equal weights and Mahalanobis distance (H_M). It is worth noting that the literature offers different methods for determining weights [41,73,74,75]. Maggino and Ruviglioni [74] observed that equal weights are commonly employed in many applications. Using equal weights simplifies the analysis and can be appropriate in situations where there is no clear justification for assigning different weights to the criteria.

Firstly, the entropy-based vector of weights is calculated according to Formulas (11) and (12). The resulting entropy-based vector of weights is as follows:

*W* = [0.263, 0.072, 0.011, 0.584, 0.070].

The ideal based on max and min values (see Formula (4)) has the form:

$$I^{+}=[2.40,\;62.60,\;100.00,\;34.70,\;79.18].$$

The criteria values are normalized according to Formula (14). Subsequently, the Euclidean or Mahalanobis distances between objects and the ideal object are computed using Formulas (9) or (15), respectively. Finally, the synthetic measure is determined through the application of Formula (10). The results of the variants of Hellwig’s measures are displayed in Table 4. 

From Table 4, we can observe significant disparities in the realization of SDG 4 of the 2030 Agenda by the EU countries in the field of education in 2021. The results indicate that the correlation between the criteria related to the choice of distance measure (Euclidean or Mahalanobis) and the system of weights (equal or entropy-based) has a significant impact on the ranking of the EU countries obtained through various versions of Hellwig’s method. We have found that, according to the H_E measure, the countries characterized by the highest positions were Slovenia with a value of 0.581, the Netherlands with 0.565, and Ireland with 0.511. Meanwhile, those with the lowest positions were Bulgaria with a value of 0.024, Romania with 0.025, and Hungary with 0.109. According to the H_EE measure, the three highest positions were taken by Sweden (0.698), the Netherlands (0.692), and Finland (0.681), while the lowest by Bulgaria (0.073), Romania (0.140), and Slovakia (0.164). We can observe that Slovenia (0.615), Sweden (0.446), and the Netherlands (0.425) secured the top three positions according to the H_M measure, whereas Romania (0.059), Slovakia (0.065), and Bulgaria (0.092) occupied the bottom positions. Finally, Sweden (0.635), Slovenia (0.633), and the Netherlands (0.590) claimed the top three positions based on the H_EM measure. In contrast, Bulgaria (0.099), Slovakia (0.118), and Hungary (0.130) occupied the lowest positions.

**Figure 6:**
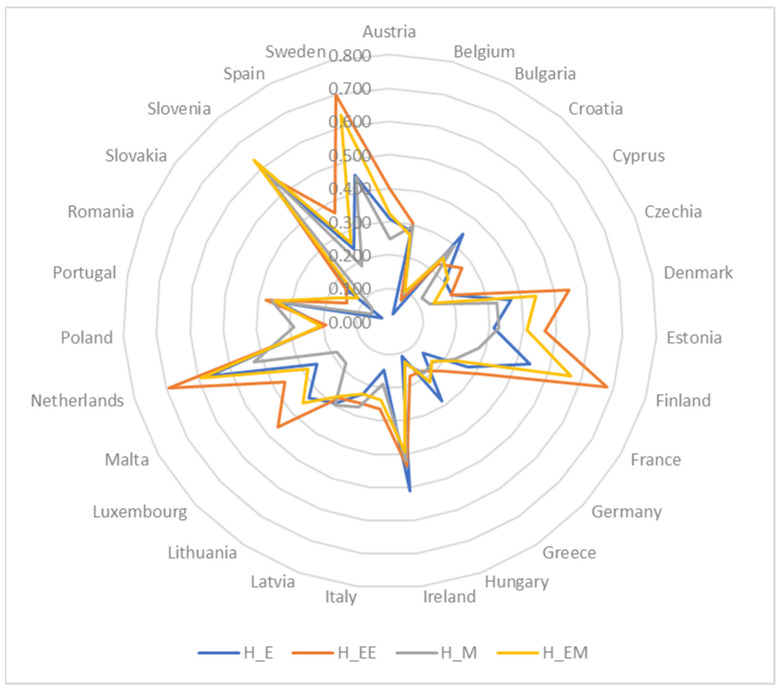
Comparison of rankings obtained through variants of Hellwig’s method.

**Figure 7:**
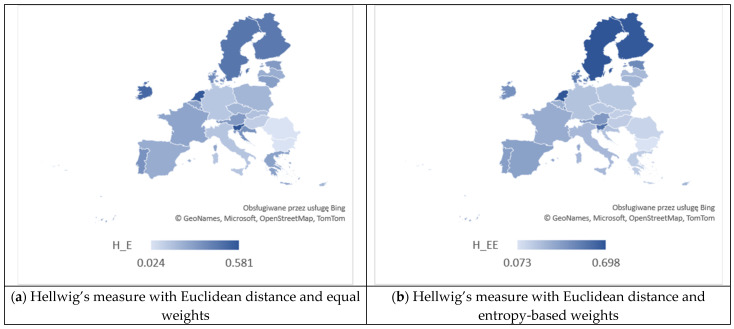
The spatial diversity in EU countries in the realization of SDG 4 with respect to values of Hellwig’s measures with Euclidean distance. Source: own evaluation using MS Excel.

**Figure 8:**
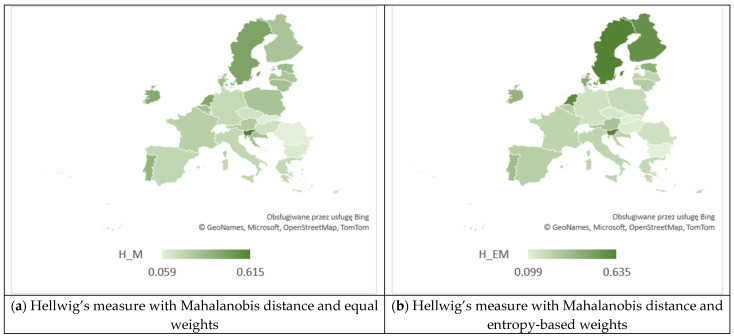
The spatial diversity in EU countries in the realization of SDG 4 with respect to values of Hellwig’s measures with Mahalanobis distance. Source: own evaluation using MS Excel.

Figure 6 provides a visual representation and comparison of the outcomes derived from Hellwig’s measures, while Figure 7 and Figure 8 depict the visualization of regional disparities based on the obtained results, confirming variability among the EU countries. The dispersions for the four applied methods are as follows: 0.557 (for H_E), 0.625 (for H_EE), 0.556 (for H_M), and 0.536 (for H_EM). This implies that no single country excelled or lagged in all criteria. For example, significant differences in individual rankings based on criteria were observed for Greece, which ranked 3rd in terms of $C_{1}$ (early leavers from education and training) and 27th in terms of $C_{3}$ (participation in early childhood education). Croatia also exhibited notable differences, securing the 1st position in terms of $C_{1}$ and the 24th position in terms of $C_{3}$. Spain demonstrated considerable variations, ranking 26th in terms of $C_{1}$ and 6th in terms of both $C_{3}$ and $C_{5}$. Likewise, Lithuania showed differences, ranking 4th in terms of $C_{2}$ and 23rd in terms of $C_{5}$.

Pearson coefficients for Hellwig’s measures and Spearman coefficients for the rank-orderings obtained by the four Hellwig’s methods are presented in Table 5 and Table 6, respectively. The strongest Pearson correlation (0.964) was noted between the H_EM and H_EE measures. Conversely, the weakest Pearson correlation (0.726) was identified between the H_EE and H_M measures.

In the examination of the EU countries’ standings within the comprehensive classification obtained through various Hellwig’s procedures, it is noteworthy that certain countries experienced improvement, while others witnessed a decline in their rankings.

The differences in the values of H_EE vs. H_E were a result of adopting a different weight system (entropy-based vs. equal weights) while assuming the independence of the criteria and using Euclidean distance. When comparing the values of H_EE vs. H_E, three countries demonstrated the most significant improvement in their values: Sweden (0.246), Finland (0.242), and Denmark (0.177), resulting in an increase in their respective rankings by 3, 2, and 1 positions. Conversely, the highest decreases in the value were observed for Croatia (0.118) and Greece (0.108), leading to a decrease in their ranking by 12 and 10 positions.

These alterations are largely attributed to the significant impact of criterion $C_{4}$ (adult participation in learning in the past four weeks), which carries a weight of 0.584 in the H_EE value due to application of entropy weights. Concurrently, the impact of criteria $C_{2}$ (tertiary educational attainment), $C_{3}$ (participation in early childhood education), and $C_{5}$ (share of individuals having at least basic digital skills) decreases significantly, as their weights are much smaller than 0.1. The fact that Croatia ranks low, 23rd place, in criterion $C_{4}$ significantly worsens its position in the ranking of H_EE. A comparable situation arises for Greece, attaining the 13th spot in the H_E ranking but slipping to the 23rd position in the H_EE ranking (a decline of 10 places). Notably, Greece holds the 26th position in criterion $C_{4}$, placing it second to last in the overall ranking. Analyzing the positions of the countries whose ranking improved after implementing entropy-based weights, it can be observed that they held top positions in criterion $C_{4}$. This led to a significant increase in the values of the H_EE measure compared to H_E. 

The disparities in the values of H_EM vs. H_M arise from the utilization of different systems of weights (entropy-based vs. equal weights) while taking into account dependencies between criteria by implementing the Mahalanobis distance measure. When comparing the H_EM with H_M values for three countries Finland (0.286), Sweden (0.189), and Luxemburg (0.176), the most significant improvements were observed. This translated to an enhancement in their respective rankings by 8, 1, and 11 positions. In contrast, notable decreases in values were identified in Poland (0.079) and Croatia (0.049), resulting in a reduction in their rankings by 10 and 6 positions, respectively. The improvement or deterioration of positions in the rankings obtained using the Hellwig’s method with Mahalanobis distance and equal or entropy-based weights, similar to the case of methods with Euclidean distance (H_E and H_EE), was primarily influenced by criterion $C_{4}$, which held the greatest weight. 

The differences in the values of H_M vs. H_E stem from the utilization of different distance measures (Mahalanobis vs. Euclidean) while maintaining equal weights in the analysis. When comparing the values of H_M with H_E for two countries Bulgaria (0.068) and Germany (0.041), the most improvement were observed, translating to an improvement in their respective rankings by 2 and 3 positions. In contrast, the most substantial declines in values were noted in Finland (0.159), Luxembourg (0.153), and the Netherlands (0.140), leading to a decrease in their rankings by 7, 10, and 1 positions, respectively. Undoubtedly, all these changes were influenced by the dependencies between the criteria.

The disparities in the values of H_EM vs. H_EE arise from the utilization of different distance measures (Mahalanobis vs. Euclidean) while maintaining entropy-based weights in the analysis. When comparing the H_EM with H_EE values for two countries Slovenia (0.058) and Greece (0.042), the most significant improvements were observed. This translated to an enhancement in their rankings by 2 and 4 positions. In contrast, notable decreases in values were identified in Spain (0.103) and the Netherlands (0.103), resulting in a reduction in their rankings by 3 and 1 positions, respectively. However, it is worth noting that in each of these cases, the differences in the values of Hellwig’s measures were very small.

Finally, in answering research questions 1 and 2, we can conclude that the distance measure and weighting method chosen affected the resulting rankings, which were confirmed with the Pearson and the Spearman rank correlation coefficients. We can observe that in the Hellwig’s method, which does not consider the interaction between the criteria, there is a tendency to overestimate values for high-scoring countries. Conversely, low-scoring countries often underestimated values compared to their counterparts in the Hellwig’s method based on the Mahalanobis distance. It is worth noting that these outcomes align with previous research findings [45,76]. 

## 4. Conclusions

This article proposes the H_EM method as an extension of the Hellwig’s method to address the subjectivity in criteria evaluation and the interdependence among criteria. To handle the unknown information about criteria weights, an entropy-based method for determining criteria weights is implemented. The Euclidean distance is replaced with the Mahalanobis distance to capture the impact of the correlation between criteria. The usability of the H_EM method is described in the context of evaluating sustainable development in the education area. The comparative analysis conducted in this paper reveals the effectiveness of the proposed approach. The results demonstrate how the presence of correlation between criteria leads to differences in the rankings of the EU countries obtained by the Hellwig’s method with the Mahalanobis distance and the Hellwig’s method with the Euclidean distance. Furthermore, the results highlight the impact of the system of weights (equal or entropy-based) on the final ranking of countries.

The classical Hellwig method has been effectively used in the area of sustainable development, for example, in sustainable agriculture [77], sustainable production [78], sustainable city [79], and sustainable economy [80], among others. The practical verification of our proposed extended Hellwig approach was conducted to assess sustainable educational development. This practical verification demonstrated that the proposed method can effectively address complex decision-making problems in the real world.

In summary, this article contributes in the following ways:

1. An extended version of the Hellwig’s method has been introduced, which takes into account the interdependencies among criteria and uncertainty about criteria weight importance. This allows for adapting the method’s framework to better handle real-life situations where criteria are interconnected, and weights are unknown.
2. The Mahalanobis distance has been employed to compute the distances between objects and the ideal object, allowing for a more accurate representation of the criteria interdependencies and their impact on the decision-making process.
3. Entropy-based weighting has been applied to objectively determine the relative importance of criteria and their contributions to the decision-making process. Additionally, a normalization formula tailored to the specific problem under investigation has been selected.
4. The results of the use of the extended Hellwig’s method have been compared with those of other Hellwig’s approaches that assume criteria independence and/or equal weight systems.
5. The studies demonstrate that the extended Hellwig’s method can be effectively applied to issues related to sustainable development. This method is better suited for practical applications, particularly when strong correlations among criteria are observed, in contrast to the classical Hellwig’s approach.

In conclusion, the advantages and limitations of those methods are shown in Table 7. Let us recall that all of them allow for evaluation and rank-ordering alternatives with respect to the set of criteria. In general, the decision-maker can choose one of the four presented variants of the Hellwig method that will be most suitable for the considered decision problem.

In the future, we aim to apply the proposed methods to address the real-life multi-criteria problems. Subsequent research will also focus on evaluating the sensitivity of the H_EM measure to other weighting criteria systems available in the literature. Additionally, the sensitivity of the H_EM measure to other distance functions and methods of establishing reference point coordinates will be tested. We will also use the proposed method to assess the implementation of the Sustainable Development Goals of the other 2030 Agenda by the European Union countries in areas other than education over several years.

## Figures and Tables

**Table 1:** Indicators measuring progress towards SDG 4 in EU in 2021.

Indicator	Criterion Type
$C_{1}:$ Early leavers from education and training (%) [sdg_04_10a]	Cost
$C_{2}$: Tertiary educational attainment (%) [sdg_04_20]	Benefit
$C_{3}$: Participation in early childhood education (%) [sdg_04_31]	Benefit
$C_{4}$: Adult participation in learning in the past four weeks (%) [sdg_04_60] (*)	Benefit
$C_{5}$: Share of individuals having at least basic digital skills (%) [sdg_04_70]	Benefit

Source: Eurostat [SDG 4] [72]. (*) data for Greece were estimated.

**Table 2:** The descriptive statistics for SDG 4 indicators in 2021.

Descriptive Statistics	$C_{1}$	$C_{2}$	$C_{3}$	$C_{4}$	$C_{5}$
Min	2.40	23.30	68.80	1.80	27.82
Max	15.30	62.60	100.00	34.70	79.18
Mean	8.24	44.58	89.22	12.65	56.29
Standard deviation	3.37	9.68	7.50	8.19	11.88
Coefficient of variation	40.84	21.72	8.40	64.73	21.10

**Table 3:** Pearson correlations between criteria.

Pearson Coefficient	$C_{1}$	$C_{2}$	$C_{3}$	$C_{4}$	$C_{5}$
$C_{1}$	1.000				
$C_{2}$	−0.437 *	1.000			
$C_{3}$	0.037	0.452 *	1.000		
$C_{4}$	−0.075	0.411 *	0.506 *	1.000	
$C_{5}$	−0.383 *	0.520 *	0.393 *	0.706 *	1.000

* p<0.05.

**Table 4:** The values and rank-ordering of EU countries obtained by the variants of Hellwig’s measures.

Country	H_E	Rank H_E	H_EE	Rank H_EE	H_M	Rank H_M	H_EM	Rank H_EM
Austria	0.306	11	0.396	9	0.248	14	0.321	10
Belgium	0.279	14	0.303	14	0.295	10	0.265	13
Bulgaria	0.024	27	0.073	27	0.092	25	0.099	27
Croatia	0.343	7	0.225	19	0.297	9	0.248	15
Cyprus	0.205	19	0.270	16	0.118	24	0.216	20
Czech Republic	0.202	20	0.198	21	0.132	23	0.144	24
Denmark	0.368	6	0.545	5	0.326	7	0.445	5
Estonia	0.313	10	0.467	6	0.327	6	0.412	6
Finland	0.439	5	0.681	3	0.280	12	0.566	4
France	0.271	15	0.314	13	0.225	15	0.237	17
Germany	0.136	24	0.214	20	0.176	21	0.173	23
Greece	0.285	13	0.176	23	0.184	18	0.218	19
Hungary	0.109	25	0.173	24	0.137	22	0.130	25
Ireland	0.511	3	0.436	8	0.422	4	0.395	7
Italy	0.145	23	0.265	17	0.189	16	0.238	16
Latvia	0.231	18	0.260	18	0.273	13	0.232	18
Lithuania	0.294	12	0.276	15	0.299	8	0.269	12
Luxembourg	0.335	9	0.460	7	0.182	19	0.358	8
Malta	0.252	16	0.364	12	0.182	20	0.284	11
Netherlands	0.565	2	0.692	2	0.425	3	0.590	3
Poland	0.201	21	0.192	22	0.287	11	0.208	21
Portugal	0.340	8	0.376	10	0.360	5	0.350	9
Romania	0.025	26	0.140	26	0.059	27	0.175	22
Slovakia	0.157	22	0.164	25	0.065	26	0.118	26
Slovenia	0.581	1	0.575	4	0.615	1	0.633	2
Spain	0.242	17	0.366	11	0.187	17	0.262	14
Sweden	0.452	4	0.698	1	0.446	2	0.635	1

Source: authors’ compilation.

**Table 5:** Pearson coefficients between Hellwig’s measures.

Pearson Coefficient	H_E	H_EE	H_M	H_EM
H_E	1.000			
H_EE	0.855 *	1.000		
H_M	0.878 *	0.726 *	1.000	
H_EM	0.891 *	0.964 *	0.835 *	1.000

* p<0.05.

**Table 6:** Spearman coefficients between rankings obtained by four Hellwig’s measures.

Spearman Coefficient	Rank H_E	Rank H_EE	Rank H_M	Rank H_EM
Rank H_E	1.000			
Rank H_EE	0.860 *	1.000		
Rank H_M	0.843 *	0.748 *	1.000	
Rank H_EM	0.913 *	0.960 *	0.825 *	1.000

* p<0.05.

**Table 7:** Advantages and limitations of variants of Hellwig’s method.

Methods	Advantages	Limitations
H_E	Rational, easy, and understandable computation. Calculation distances from each alternative to ideal one. Using equal weights simplifies the analysis and can be appropriate in situations where there is no clear justification for assigning different weights to the criteria.	The use of equal weights may not be appropriate in situations where there is justification or information supporting the assignment of different weights to the criteria. The assumption of independence among criteria is made, and the correlation between criteria cannot be taken into account.
H_EE	Rational, easy, and understandable computation. Calculation distances from each alternative to ideal one. An objective method for determining weights based on information content is employed. The entropy-based method is straightforward and uncomplicated, utilizing only information provided by criteria.	The entropy-based weight system is implemented. Subjective weight determination cannot be taken into account. The assumption of independence among criteria is made, and the correlation between criteria cannot be taken into account.
H_M	Rational, easy, and understandable computation. Calculation distances from each alternative to ideal one. Using equal weights simplifies the analysis and can be appropriate in situations where there is no clear justification for assigning different weights to the criteria. The interdependencies among criteria are taken into account.	The system of equal weight is not appropriate in situations where there are some justifications or information for assigning different weights to the criteria. The non-linear correlation between criteria cannot be taken into account.
H_EM	Rational, easy, and understandable computation. Calculation distances from each alternative to ideal one. An objective method for determining weights based on information content is employed. The entropy-based method is straightforward and uncomplicated, utilizing only information provided by criteria. The interdependences among criteria are taken into account.	The entropy-based weight system is implemented. Subjective weight determination cannot be taken into account. The non-linear correlation between criteria cannot be taken into account.

## Data Availability

Data are contained within the article.

## Abbreviations

The following abbreviations are used in this manuscript:

H_E	Hellwig’s method with equal weights and Euclidean distance
H_EE	Hellwig’s method with entropy-based weights and Euclidean distance
H_M	Hellwig’s method with equal weights and Mahalanobis distance
H_MM	Hellwig’s method with entropy-based weights and Mahalanobis distance
DM	Decision maker
TODIM	An acronym in Portuguese for Interactive and Multi-criteria Decision Making
MCDM	Multi-criteria decision-making
SDG	Sustainable Development Goal
TOPSIS	Technique for Ordering Preferences by Similarity to Ideal Solution
**Notation**	
The most important notations used in this manuscript:	
$A_{1},A_{2},\ldots ,A_{m}$	Alternatives
$C_{1},C_{2},\ldots ,C_{n}$	Decision criteria
$D=\left[x_{ij}\right]$	Decision matrix
$W=\left[w_{1},\ldots ,w_{n}\right]$	Vector of weights
$I=\left[x_{1}^{+},\ldots ,x_{n}^{+}\right]$	Ideal (pattern of development)
$\bar{D}=\left[{\bar{x}}_{ij}\right]$	Normalized matrix
$\tilde{D}=\left[{\tilde{x}}_{ij}\right]$	Weighted normalized matrix
$d_{i0}(A_{i},I)$	The distances of i-th alternative $A_{i}$ from the ideal I
$H_{i}$	Hellwig’s measure for the i-th alternative
$E_{j}$	Extended and normalized entropy
M(x,y)	Weighted Mahalanobis distance between points x and y
